# Transverse colon volvulus presenting as bowel obstruction: a case report

**DOI:** 10.1186/s13256-019-2080-1

**Published:** 2019-05-25

**Authors:** Hamza Hasnaoui, Faouzi Laytimi, Yusuf Elfellah, Ouadii Mouaqit, El Bachir Benjelloun, Abdelmalek Ousadden, Khalid Ait Taleb, Hicham El bouhaddouti

**Affiliations:** 1 Visceral Surgery Department A, CHU Hassan II, Fez, Morocco; 2 Faculty of Medicine and Pharmacy, Sidi Mohamed Ben Abdellah University of Fez, Fez, Morocco

**Keywords:** Transverse colon, Volvulus, Bowel obstruction

## Abstract

**Background:**

Transverse colon volvulus is an uncommon cause of bowel obstruction. The total number of cases reported in the literature is 100. It constitutes a surgical emergency since it can lead to bowel infarction, peritonitis, and death if not diagnosed at once. It seemed appropriate to report this case that was treated at the Department of Visceral Surgery A, University Hospital Center Hassan II of Fez in Morocco.

**Case presentation:**

We report a rare case of transverse colon volvulus in a 42-year-old Arabic man, with no particular history, who presented to our emergency department with a 5-day history of constipation, progressive abdominal pain, nausea, and vomiting. His last bowel movement had been 3 days ago. Abdominal radiography showed a large bowel obstruction with a “U-shaped” loop in the left upper abdomen. Abdominal computed tomography was not performed because of impaired renal function.

He was operated on urgently after conditioning and the diagnosis of a transverse colon volvulus was done intraoperatively. Rotated in a 360° clockwise direction on its mesentery, the bowel was intact without signs of ischemia. An extended right hemicolectomy was carried out with end-to-side ileocolic anastomosis.

Through this case, we will try to discuss its physiopathology, etiologies, diagnosis, and management in emergencies.

**Conclusion:**

This case is unusual because no etiological factor has been found. Its diagnosis can be difficult and management effectiveness remains controversial.

It is important to highlight this case and those of the literature, as many surgeons may have never seen a case of transverse colon volvulus. Volvulus of the transverse colon may therefore not be considered in the differential diagnosis of recurrent intermittent abdominal pain or acute intestinal obstruction.

Prompt recognition with emergency intervention constitutes the key to a successful outcome.

## Introduction

Transverse colon volvulus is an uncommon cause of bowel obstruction. It constitutes a surgical emergency since it can lead to bowel infarction, peritonitis, and death if not diagnosed at once. Abdominal computed tomography (CT), performed on an emergency basis, can help to diagnose this obstruction before surgery and help in the selection of a therapeutic approach. It seemed appropriate to report this case that was treated at the Department of Visceral Surgery A, University Hospital Center (CHU) Hassan II of Fez in Morocco.

## Case presentation

A 42-year-old Arabic man presented to general surgery emergency with a 5-day history of constipation, progressive abdominal pain, nausea, and vomiting. His last bowel movement had been 3 days ago. There was no significant past medical history, particularly of chronic constipation, psychiatric disease, or abdominal surgery.

**Fig. 1:**
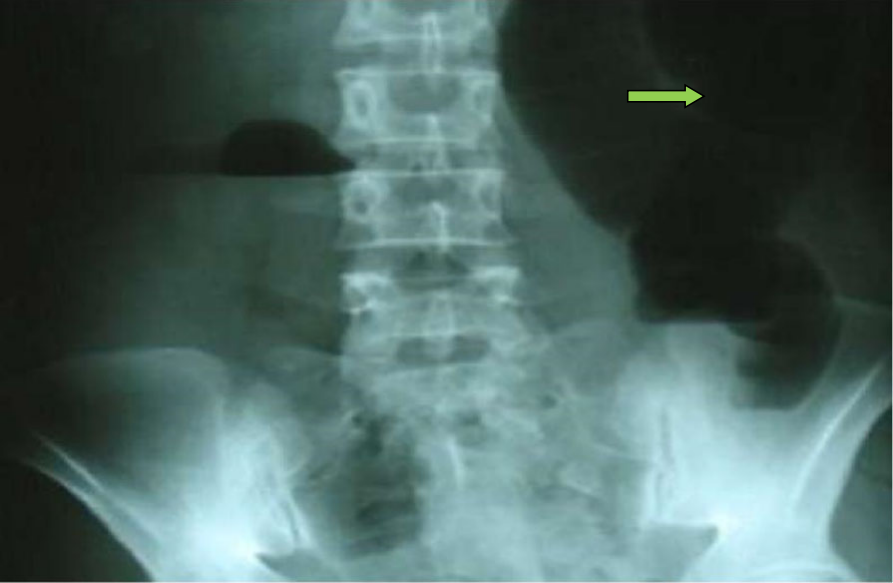
Abdominal X-ray showing (arrow) a dilated colon with a “U-shaped” loop in the left upper abdomen

On examination, his vital signs were: temperature 37.5 °C, pulse 115/minute, respiratory rate 26/minute, and blood pressure 90/60 mmHg. An abdominal examination revealed a massive distension of his abdomen without signs of peritonitis. His abdomen was tympanic to percussion. There were no umbilical or groin hernias. A digital rectal examination demonstrated an empty rectal vault without intraluminal masses. An abdominal X-ray revealed a large bowel obstruction with a “U-shaped” loop in the left upper abdomen (Fig. 1).

Blood investigations showed leukocytosis at 12.0 × 10^9^/L, C-reactive protein (CRP) at 34 mg/l, and serum sodium and potassium levels were within normal limits.

An abdominal CT could not be done due to functional renal failure.

**Fig. 2:**
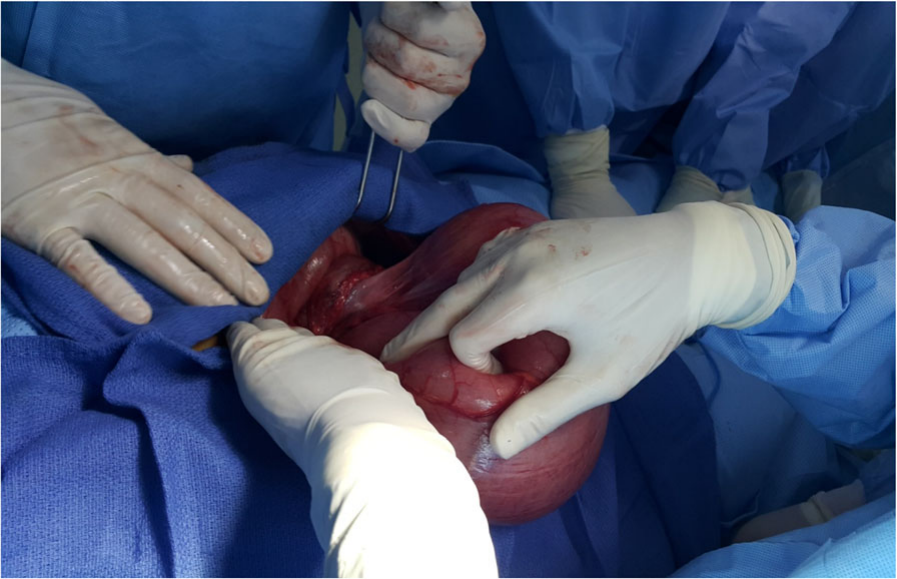
Gross operative view of volvulus of the transverse colon

**Fig. 3:**
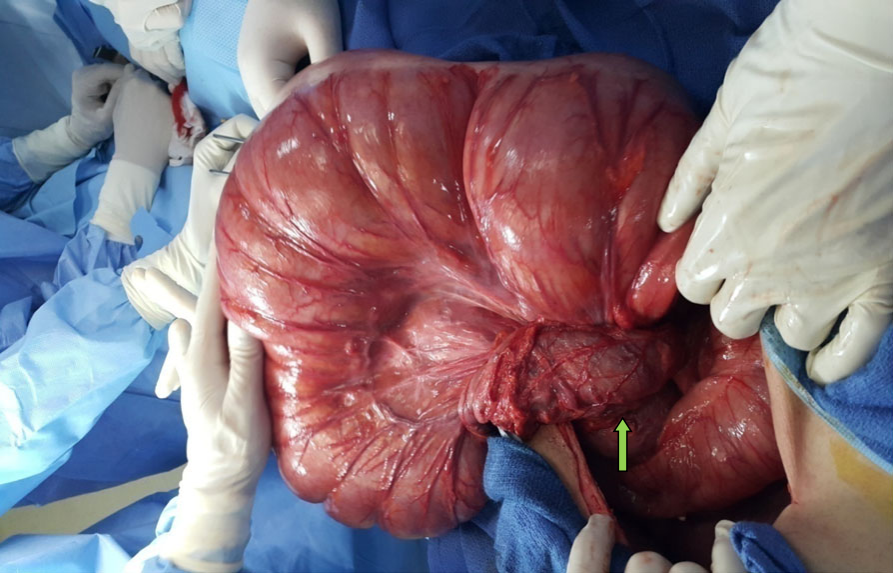
Intraoperative view demonstrating volvulus of transverse colon. The point of twist (arrow) was located in the left upper quadrant of the abdomen

**Fig. 4:**
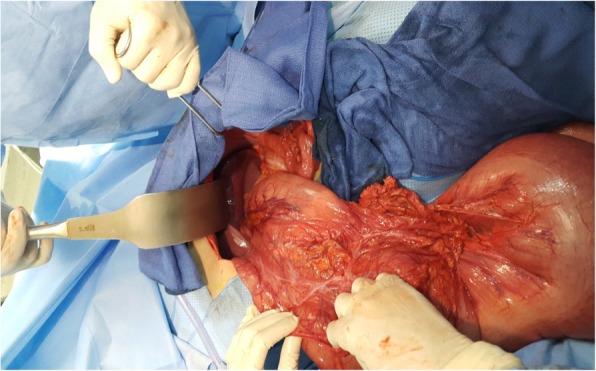
Intraoperative view demonstrating volvulus of transverse colon without signs of ischemia

After initial resuscitation with intravenously administered fluids, analgesics, and antibiotics, a decision was taken to proceed with an emergency laparotomy. Intraoperative findings (Fig. 2) were of a transverse colon volvulus rotated in a 360° clockwise direction on its mesentery. The point of twist was found in the left upper quadrant (Fig. 3). The bowel was intact without signs of ischemia (Fig. 4). A significant disparity in the size of the obstructed proximal and collapsed distal colon to the site of the volvulus was noticed. The transverse colon was mobile and increased in length. The volvulus was delivered into the incision and detorsed. An extended right hemicolectomy was carried out with end-to-side ileocolic anastomosis.

Our patient’s postoperative course was uneventful. He was discharged from hospital 6 days following admission. On histologic examination, the appearance was consistent with a subacute progressive volvulus of the transverse colon. No acute inflammation, infarction, granulomas, dysplasia, malignancy, or vascular abnormality was noticed.

## Discussion

Volvulus of the transverse colon was first described in 1932 by the Finnish surgeon Kallio [1]; it constitutes approximately 5% of all causes of bowel obstruction [2]. Each segment of the colon may get rotated if it has a long loose mesentery, which narrows at its base [3]. The anatomical build causes 60–80% of cases to be related to the sigmoid colon, followed by the cecum 20–40% [2]. Volvulus of the transverse colon occurs extremely rarely. Until now, less than 100 patients were described to have such a diagnosis [4].

Short mesentery of the transverse colon and fixed hepatic and splenic flexure are undoubtedly factors that prevent it. Among predisposing factors, we can identify earlier surgical procedures causing concrescence or bowel translocation, cancer, pregnancies, and congenital defects such as intestinal malrotation with an imperfect fixation of the posterior abdominal wall [4–6]. Moreover, chronic constipation seems to be associated with the occurrence of transverse colon volvulus by causing its excessive elongation [7]. Yaseen *et al*. described the coexistence of *Clostridium difficile* infection with volvulus, postulating the participation of mucositis in the pathogenesis [8].

The twisting of the intestine around the mesenteric axis is connected with closure of its loop, retention of the venous outflow because of the compression of the vessel, and, possibly, impaired arterial flow.

Given the clinical picture and morphological transformations, our patient had no past medical history of chronic constipation, psychiatric disease, neurologic disease, or abdominal surgery but presented with subacute transverse volvulus. A progressive onset of the symptoms can delay the diagnosis and the treatment thus resulting in progression to the acute fulminating type with bowel infarction, peritonitis, and even death [4].

Subacute onset is characterized by massive abdominal distension in the setting of mild abdominal pain without rebound tenderness, nausea, or vomiting [9]. The leukocyte count is normal secondary to a lack of ischemia at early stages. Patients with the acute fulminating type of presentation have a sudden onset of severe abdominal pain, rebound tenderness, vomiting, little distension, and rapid clinical deterioration. Bowel sounds are initially hyperactive becoming absent later on [9, 10].

The diagnosis of volvulus of the transverse colon before surgery is rarely observed. There are no characteristic radiographic features, as in the case of volvulus of the sigmoid colon [9]. Some authors suggested that the presence of a distended colon with two levels of fluid in the epigastrium in X-ray may suggest the diagnosis [11]. It is usually made intraoperatively. In the subacute type, the achievement of an early diagnosis through CT is advised [10].

Lower gastrointestinal series of contrast that produces the image of a bird’s beak around the transverse colon can be much more helpful, but, in the case of acute symptoms, the performance of this examination should not delay the commencement of urgent surgery [4]. It is worth mentioning that the co-occurrence of this disease with Chilaiditi syndrome has been reported in the literature several times. This syndrome is characterized as a displacement of the hepatic flexure of the colon between the liver and the diaphragm. It is detected on X-ray and often confused with air in the peritoneum [12].

In contrast to volvulus of the sigmoid colon and cecum, an attempt at endoscopic decompression and drainage of the colon is not recommended mainly due to the high probability of necrosis [13]. Resection constitutes the treatment of choice to prevent recurrence [4, 9]. In fact, detorsion alone or associated with colopexy has a higher rate of recurrence than resection [4, 9]. The incidence of recurrent volvulus after previous resection and primary anastomosis varies between 22 and 36% [14]. Therefore, some authors recommend considering a subtotal colectomy in the presence of a megacolon, instead of partial resection of the involved bowel segment [14]. This resection is carried out with or without primary anastomosis dependent on the aspect of the colon, the existence or not of peritonitis, and the state of health of the patient.

Our patient had an extensive right hemicolectomy with end-to-side ileocolic anastomosis. His postoperative course was uneventful.

In the case of volvulus of the transverse colon, the mortality rate is 33%, which is much higher than the mortality rate recorded for volvulus of the sigmoid colon or cecum, which is 21% and 10%, respectively [4].

## Conclusion

Transverse colon volvulus is a rare cause of bowel obstruction in our daily practice. Its diagnosis is challenging. Prompt recognition with emergency intervention constitutes the key to a successful outcome.
